# Recent Developments in the Use of Glyconanoparticles and Related Quantum Dots for the Detection of Lectins, Viruses, Bacteria and Cancer Cells

**DOI:** 10.3389/fchem.2021.668509

**Published:** 2021-07-19

**Authors:** Pedro J. Hernando, Simone Dedola, María J. Marín, Robert A. Field

**Affiliations:** ^1^ Iceni Diagnostics Ltd., Norwich Research Park Innovation Centre, Norwich, United Kingdom; ^2^ Quadram Institute Bioscience, Norwich, United Kingdom; ^3^ School of Chemistry, University of East Anglia, Norwich, United Kingdom; ^4^ Department of Chemistry, Manchester Institute of Biotechnology, University of Manchester, Manchester, United Kingdom

**Keywords:** glyconanoparticles, gold nanoparticles, rapid diagnostics, pathogen detection, cancer imaging, glycobiology

## Abstract

Carbohydrate-coated nanoparticles—glyconanoparticles—are finding increased interest as tools in biomedicine. This compilation, mainly covering the past five years, comprises the use of gold, silver and ferrite (magnetic) nanoparticles, silicon-based and cadmium-based quantum dots. Applications in the detection of lectins/protein toxins, viruses and bacteria are covered, as well as advances in detection of cancer cells. The role of the carbohydrate moieties in stabilising nanoparticles and providing selectivity in bioassays is discussed, the issue of cytotoxicity encountered in some systems, especially semiconductor quantum dots, is also considered. Efforts to overcome the latter problem by using other types of nanoparticles, based on gold or silicon, are also presented.

## Introduction

The use of nanoparticles (NPs) as biomedical tools has developed at pace in recent years, with NPs functionalised with carbohydrates (glyconanoparticles) emerging in diagnostics and cell imaging. Since the first report of glyconanoparticles (de la Fuente et al., 2001), interest in these materials has risen considerably. The main approaches to the preparation and early applications of glyconanoparticles have been reviewed previously (de la Fuente and Penadés, 2006; Marradi et al., 2013; Chen et al., 2014), as has the application of glyconanoparticles in biomedicine (Dosekova et al., 2017; Kveton et al., 2020), the use of magnetic glyconanoparticles in biosensing (Fratila et al., 2016), and glyconanoparticles for the detection of cancer cells and the early diagnosis of cancer (Hockl et al., 2016; Torres-Pérez et al., 2020).

The first examples of glyconanoparticles focused on gold, silver, and iron oxide systems. The impact of the size and shape of the nanoparticle, as well as the density of carbohydrates on the surface of the nanoparticle and the importance of the tether employed for the functionalisation, have been studied and reviewed (Compostella et al., 2017). Key conclusions are that, for certain applications, nanoparticles with larger size present flatter surfaces, enhancing carbohydrate-target interactions (Chien et al., 2008); the shape of the particle (rods vs. spheres) can also impact on the detection limit of *Escherichia* coli-glyconanoparticle interactions, for instance (Chaudhary et al., 2015). Nanoparticles have a high surface to volume ratio, enabling multivalent ligand presentation, which has been widely exploited to overcome the inherently weak nature of carbohydrate-protein interactions (Lundquist and Toone, 2002). However, a too high density of the carbohydrate on the surface can hamper accessibility of the glycan to protein partners—a challenge that can be addressed through longer, more flexible tethers (Marradi et al., 2013; Compostella et al., 2017).

More recent efforts has seen the exploration of semi-conductor nanoparticles (quantum dots–QDs) in the field of diagnostics (Medintz et al., 2005; Michalet et al., 2005). In the range of 1–10 nm, cadmium-based QDs are inherently fluorescent (Alivisatos, 1996; Bruchez et al., 1998; Li et al., 2015). The evident cytotoxicity of semi-conductor QDs prompted the exploration of the more biocompatible silicon quantum dots (SiQDs) (Robidillo and Veinot, 2020), which had no effect on cell viability in Shewanella oneidensis and Bacillus subtilis (Pramanik et al., 2018). A recent comprehensive review of QDs, glyco-QDs and their synthesis and applications in biosensing is suggested (Marradi et al., 2021).

**FIGURE 1:**
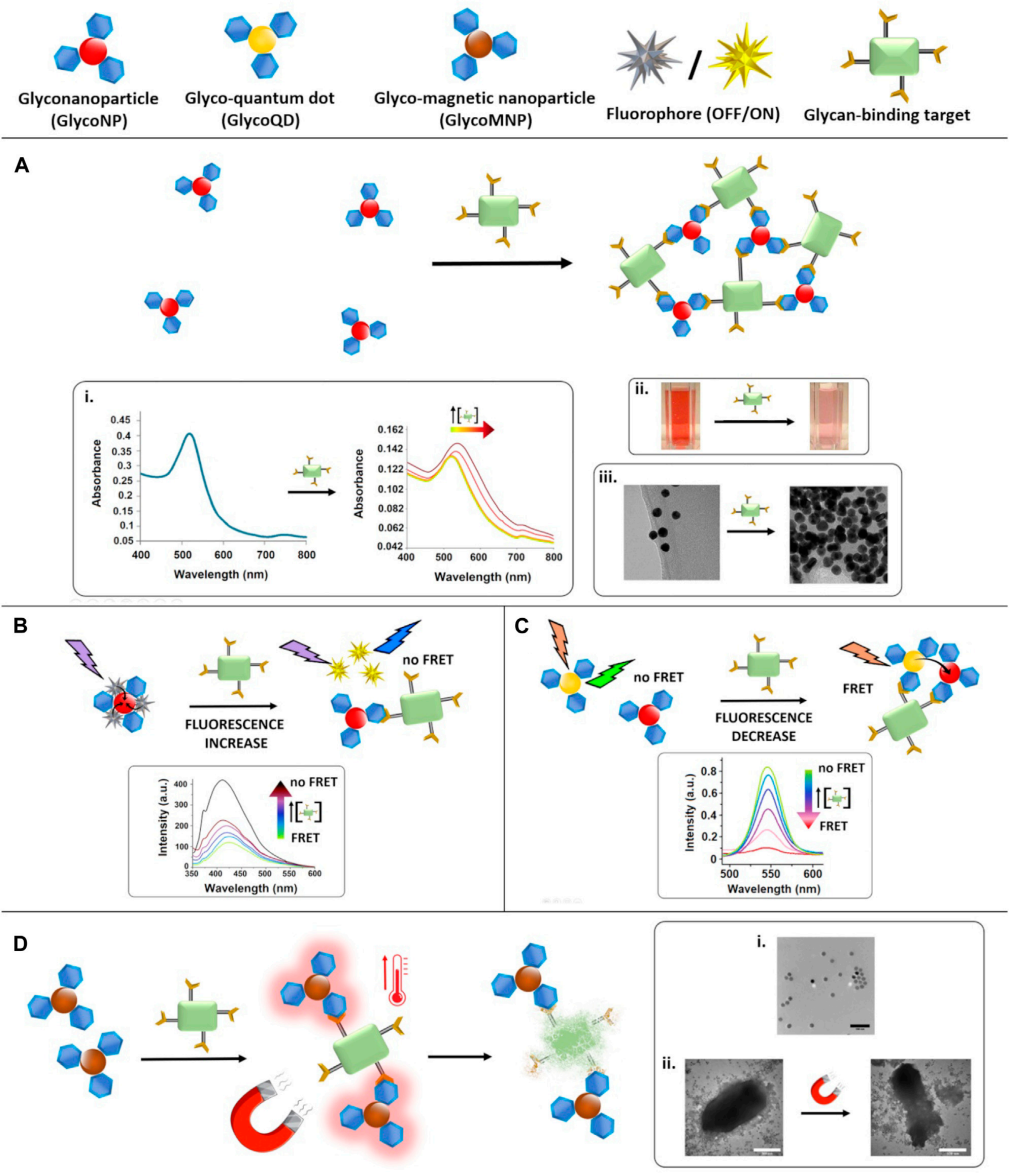
Glyconanoparticle-based assays for the detection of carbohydrate-binding targets. **(A)**: the presence of the target reduces the interparticle distance as result of the aggregation of the NPs. Insets show the impact on i) plasmonic (reproduced from Poonthiyil et al., 2015a with permission from the Royal Society of Chemistry), ii) colorimetric (reproduced from Marín et al., 2015 with permission from the Royal Society of Chemistry) and iii) TEM (reproduced from Poonthiyil et al., 2015a with permission from the Royal Society of Chemistry) detection. **(B)**: FRET-based assay–a fluorophore is quenched upon attachment to a glycoNP and the fluorescence is recoverd in the presence of the target. Inset shows the fluorescence recovery observed by fluorescence spectroscopy (adapted with permission from Ajish et al., 2020. Copyright 2020 American Chemical Society). **(C)**: FRET-based assay–glycoQDs are quenched in the presence of the target, due to a decrease in the distance with equally functionalised glycoNPs. Inset shows the decrease in fluorescence emission intensity of the QDs (adapted from Zhang et al., 2018 with permission from Taylor & Francis Ltd) with increasing concentrations of the target. **(D)**: MagMED-based assay to study bacteria death using MNPs and an alternating magnetic field. Insets show i) a TEM image of the NPs dispersion, and ii) TEM of the target in the prence of the MNPs before and after treatment with the magnetic field which causes bacterial death consequently to the temperature increase (adapted from Raval et al., 2017 with permission from John Wiley and Sons).

To date, glyconanoparticle-based detection assays have proven effective for lectin detection, including those associated with viruses and bacteria. The tuneable optical properties of gold NPs (AuNPs) and silver nanoparticles (AgNPs) make them ideal candidates for use in simple colorimetric assays (Schofield et al., 2006; Marín et al., 2015). Given the pervasive nature of carbohydrates in biology (Dedola et al., 2020), and aberrant expression of carbohydrates and carbohydrate-binding receptors on the surface of some cancer cells, glyconanoparticles have also been extensively studied to target, image and treat tumours (Hockl et al., 2016; Torres-Pérez et al., 2020). Herein, we survey recent advances in these fields: key features of the systems discussed can be found in Figure 1 and Table 1.

**TABLE 1:** GlycoNPs and QDs for detection, diagnosis and imaging, indexed by particle type.

Type of particle	Target	References
Gold NPs	*Escherichia coli*	Richards et al. (2014), Qi et al. (2018), Ajish et al. (2020)
	*Pseudomonas aeruginosa*	Qi et al. (2018), Khan et al. (2019), Zhang et al. (2020)
	*Micrococcus luteus*, *Vibrio alginolyticus*, *Shewanella alginolyticus*, *Desulfovibrio desulfuricans*	Qi et al. (2018)
	Human influenza virus	Marín et al. (2013), Poonthiyil et al. (2015b), Zhang et al. (2016), Zheng et al. (2017)
	*Sambucus nigra* agglutinin (SNA)	Zhang et al. (2016)
	SARS-CoV-2 spike protein (VLPs)	Baker et al. (2020)
	DC-SIGN/R proteins	Budhadev et al. (2020)
	*Escherichia coli* enterotoxin LTB	Poonthiyil et al. (2015a)
	SIGLEC proteins	Schofield et al. (2016)
	Galectin-1	García Calavia et al. (2018)
	Lung cancer cells	Adokoh et al. (2020)
Silver NPs	*Escherichia coli*	Wang et al. (2017)
	*Cholera toxin* B subunit (CTB)	Simpson et al. (2016)
Magnetic NPs (ferrite, alumina)	*Escherichia coli*	Park et al. (2017), Raval et al. (2017)
	*Helicobacter pylori*	Park et al. (2015)
	*Mycobacterium smegmatis*	Jayawardana et al. (2015)
	Shiga-like toxin 1 (Stx1)	Kuo et al. (2015)
Silicon QDs	*Escherichia coli*	Jayawardena et al. (2013)
	*Mycobacterium smegmatis*	Jayawardana et al. (2015)
	Cancer cells	Ahire et al. (2015), Lai et al. (2016), Hsu et al. (2017), Li et al. (2020)
Cadmium QDs	DC-SIGN/R proteins	Guo et al. (2016), Guo et al. (2017)
	Plant lectins (ConA, PNA)	Ma et al. (2014)
QDs + gold NPs	*Cholera toxin* B subunit (CTB)	Ahn et al. (2016)
	Concanavalin A (ConA)	Zhang et al. (2018)

## Lectins/Protein Toxins

The plant lectin Concanavalin A (ConA) has been widely used as a model to develop carbohydrate-based lectin detection systems. The specific interaction between glucosamine-functionalised AuNPs and ConA has been reported (Di Silvio et al., 2018) to study the intracellular exchange of protein corona, confirming that specific interactions between lectins and NPs surface ligands contribute to retain the lectins on the surface of the NP. Thiolated mannosides have been used to functionalise AuNPs, as well as ZnS/CdSe QDs, for the detection of ConA (Zhang et al., 2018). In this, and several other studies, fluorescence quenching *via* Förster resonance energy transfer (FRET) was used as a switch on-off effect during the assay.

CdSe/ZnS QDs have been functionalised with quinolyl glucose (Glc) or quinolyl galactose (Gal) for the detection of ConA and peanut agglutinin (PNA), respectively (Ma et al., 2014). Both Glc and Gal systems exploited the FRET effect between the QDs and the quinone, with the fluorescence recovery achieved in the presence of the specific lectins. Other studies applied glyco-QDs for the detection of lectins with a dual-colour system based on glucose-QDs for the detection of ConA and galactose-QDs for the detection of PNA, allowing the discrimination between both lectins in the same sample (Zhang et al., 2013). More recent work combined different glyco-QDs with red, green and yellow emissions for selective detection between ConA, PNA, Pisum sativum agglutinin, wheat germ agglutinin and Ricinus communis agglutinin 120 (Wang et al., 2018).

The detection of bacterial toxins has attracted attention in diagnostic. The detection of Cholera toxin (CTB) (Schofield et al., 2007) and the detection of heat-labile enterotoxin B subunit (LTB) from *E. coli* (Poonthiyil et al., 2015a), both based on galactose-functionalised AuNPs, have been reported. In the latter, 12 nm diameter AuNPs produced the most significant shift in absorbance and the toxin was detected at a concentration of 100 nM. The detection of Shiga-like toxin 1 (Stx1), often associated with bacteria such as *E. coli* or *Shigella dysenteriae*, has been also achieved with glyconanoparticles. A systematic comparison of tether lengths and nanoparticle sizes was made using globotriose-functionalised AuNPs (Chien et al., 2008), showing that larger particles with longer tethers allowed a more efficient binding to Stx1 when they were compared to the free globotriose ligand. A different approach was adopted using a magnetic nanoparticle-based system (MNPs) (Kuo et al., 2015). Here, the glycan functionality was conveniently obtained by functionalising 30 nm particles with pigeon ovalbumin, a glycoprotein containing an oligosaccharide ligand for Stx1, namely Gal-α(1→4)-Gal-β(1→4)-GlcNAc. The assay showed specificity for Stx1 in complex matrices, where the glyconanoparticles and associated protein toxin were isolated by magnetic extraction and analysed by MALDI-ToF to confirm the presence of St×1.

Chromophores are often combined with either an enhancing or a supressing counterpart. A surface-enhanced Raman spectroscopy (SERS)-based assay for the detection of CTB was developed using silver nanoparticles presenting both PEGylated galactose and sialic acid (SA) (Simpson et al., 2016) (optimised 15:1 ratio). This assay allowed the low nM detection of the toxin in simulated freshwater samples.

A system comprising galactose-AuNPs and amine-QDs for the detection of CTB has been developed (Ahn et al., 2016). The assay was based on the inhibition of fluorescence *via* FRET, upon binding of the QDs to the AuNPs *via* hydrogen bonds formation between the amines on the QD and the hydroxyl groups of galactose-AuNPs. In the presence of CTB, the hydrogen bonds are disrupted liberating the amine-QDs and activating their fluorescence.

Selectins have attracted attention as biomarkers for the diagnosis of brain inflammation. Lewis X (Le^x^)-capped ferrite nanoparticles were designed for the selective *in vivo* targeting of such receptors (Van Kasteren et al., 2009). This work showed potential for the early diagnosis of neuropathologies such us dementia, encephalitis or Parkinson’s disease.

## Viruses

Influenza virus remains a serious global health concern, causing ca. 300,000 deaths every year (Paget et al., 2019). The affinity between hemagglutinin on the surface of the virus and sialic acid, which forms the basis of host cell adhesion and invasion during infection, has been explored for the development of rapid diagnostics. Human influenza strains preferentially bind to α2,6-sialylgalactose, while the animal viruses prefer the α2,3-linked isomer (Marín et al., 2013). A collection of AuNPs functionalised with seven different sialic acid derivatives (Zheng et al., 2017) was used to successfully detect 14 different influenza strains enabling discrimination from other respiratory viruses, such as hRSV and avian influenza virus.

A bi-antennary sialoglycopeptide extracted from egg yolk has been used to functionalise AuNPs for the colorimetric detection of human influenza virus (Poonthiyil et al., 2015b) achieving a detection limit of 71 nM and the effective detection of two H1N1 strains, A/PuertoRico/8/34 and A/New Caledonia/29/1999.

A straightforward methodology for the preparation of AuNPs coated with α2,6-sialyllactose-containing polymer has been reported by Zhang et al. (Zhang et al., 2016) and tested for aggregation with *Sambucus* nigra agglutinin and influenza virus using dynamic light scattering or transmission electron microscopy (TEM).

Since the pandemic crisis caused by COVID-19 in early 2020, efforts have been made to detect SARS-CoV2 using glyconanoparticles. A lateral flow system for the rapid detection of coronavirus spike proteins was recently reported (Baker et al., 2020). Sialic acid-linked poly-(2-hydroxyethyl acrylate) (PHEA) was used to functionalise AuNPs, and binding to the target spike proteins was evaluated by biolayer interferometry. The system was transferred into lateral flow format enabling the rapid, sensitive and selective detection of virus-like particles presenting the SARS-CoV2 spike protein.

Virus-glycan interactions have been shown to prevent virus internalisation in human cells through DC-SIGN/R receptors, which function as an entrance gate for viruses such as HIV or Ebola. AuNPs functionalised with the same high-mannose glycans present in the HIV glycoprotein gp120 (Chiodo et al., 2013) together with PEGylated α-fucosylamides (Arosio et al., 2014), both successfully interacted with the DC-SIGN receptor with a comparable efficiency. Other works have demonstrated that such virus uptake channels can be blocked by multivalent glyconanoparticles, and their efficacy can be modulated by varying the sugar density on the nanoparticle surface (Guo et al., 2016). In addition, the glyconanoparticle approach can be used to quantify binding affinity for DC-SIGN/R receptors as well as to inhibit viral cell entry (Guo et al., 2017). Extension of this work explored the interaction of mannose (Man)-functionalised AuNPs and DC-SIGN (Budhadev et al., 2020).

## Bacteria

The detection of bacterial pathogens represents an ongoing need in the field of health care. To target the *E. coli* fimbrial adhesion FimH, mannose-coated CdS QDs have been used (Mukhopadhyay et al., 2009). More recent work screened AuNPs functionalised with either mannose or glucose (Richards et al., 2014) to detect *E. coli* K-12 strain (FimH+) and using the TOP10 strain (FimH-) as negative control. The stability of the nanoparticles in solution was improved by using a PEG-3000 tether, rather than directly binding the respective thiosugar to the AuNPs.

Recently, a detection system for *E. coli* based on the fluorescent properties of glycoacrylamides (Glc-bis) has been reported (Ajish et al., 2020), where self-aggregation-induced π interactions between the acrylamide moiety installed on the glucose generate the fluorescence of the polymer (Ajish et al., 2018). The fluorescence of the glycopolymer is quenched by the AuNPs, but in the presence of a glucose-binding target the Glc-bis ligand is removed from the surface of the AuNPs, reactivating its fluorescence. This turn on-based fluorescent system afforded a simple means to detect *E. coli*.

Focusing on the same FimH target, mannose-stabilised AgNPs have been synthesised for the selective detection of *E. coli* strain O157:H7 (Wang et al., 2017). Specificity was tested against a series of bacterial strains, demonstrating that the mannose-stabilised AgNPs were specific for the O157:H7 *E. coli* strain. The AgNPs construct exhibited a dual activity resulting from the targeting role of the glycan and the bactericidal properties of silver, leading to the rapid sterilisation of an *E. coli*-contaminated sample.

Given the associated cell surface lectins (LecA, B), galactose- or fucose-functionalised AuNPs have been used to target, detect and kill *Pseudomonas aeruginosa* (Zhang et al., 2020). The functionalisation was performed *via* copper-free click chemistry between azidobutyl glycosides and a cyclooctyne-based thioctic acid linker (Karamanska et al., 2005). Successively, the nanoparticles were decorated with the antibiotic ceftazidime *via* non-covalent interaction. The antibiotic activity was studied through photo-and chemotherapy, revealing that the system was selective for *P. aeruginosa* in the presence of *E. coli* or methicillin-resistant *Staphylococcus aureus*.

Gold-coated, Mn-doped magnetite nanoparticles functionalised with mannosamine have been used to target E.*coli* (Park et al., 2017), as judged by TEM. Using a more sophisticated system, Raval et al. targeted *E. coli* with the bacteria-specific glycoconjugate GM3 [Neu5Ac(α2-3)-Gal-β(1-4)Glc-βsp] “clicked” onto the surface of magnetite nanoparticles. Anti-bacterial effects were achieved *via* magnetically-mediated energy delivery (MagMED), where heat is generated *in situ* by the application of alternating magnetic fields (Raval et al., 2017).

The modulation of binding and uptake of several types of NPs by *E. coli* using different glycans has been reported (Jayawardena et al., 2013). The carbohydrate moiety selected to stabilise the NPs (SiNPs, MNPs, Si-coated MNPs and Si-coated CdQDs) directed the specificity of the binding. Using maltoheptaose for the functionalisation of the particles remarkably improved their internalisation by the bacteria, whereas using mannose favoured the surface binding, due to the interaction of the NPs with FimH adhesins.

AuNPs functionalised with sulfated seaweed polysaccharide fucoidan showed inhibitory effects on *P. aeruginosa* growth and biofilm formation, decreasing the virulence and motility of the bacteria (Khan et al., 2019).

Fluorescent Cu/CdSQDs functionalised with glucose, stachyose or raffinose showed discrimination between different bacteria demonstrated *via* linear discrimination analysis of the fluorescence signals (Qi et al., 2018). The assay could be performed in 30 min and was able to selectively differentiate between *E. coli*, *P. aeruginosa*, *Micrococcus luteus*, *Vibrio* *alginolyticus*, *Shewanella algae* and *Desulfovibrio desulfuricans*.

A fluorescent magnetic assay to target, block or extract *Helicobacter pylori* based on fucose-containing oligosaccharides Le^a^, Le^b^ or blood group H type 1 coupled to cobalt-ferrite magnetic nanoshells has been reported (Park et al., 2015). Binding of the nanoparticles to *H. pylori* was confirmed by confocal microscopy, while incubation of the bacteria with mammalian cells in presence of these fucose-NPs prevented the adhesion of *H. pylori* to the cells.

Tuberculosis remains an important disease globally and early diagnosis represents an unmet need. A detection assay for *Mycobacterium smegmatis* has been developed (Jayawardana et al., 2015) based on silica and iron NPs. The authors assembled a small library of trehalose-stabilised NPs, using previously reported methodology (Wang et al., 2013), and demonstrated the ability of the glyconanoparticles to selectively bind *M. smegmatis* over mammalian cells.

## Cancer

The imaging of tumours and the early detection of cancer biomarkers is topical. One of the first reported application of glyco-QDs was dedicated to the study of asialoglycoprotein receptor interaction with galactose-terminated QDs in liver cancer cell line HepG2 (Kikkeri et al., 2009). Using flow cytometry, the authors demonstrated the increased uptake of galactosamine-capped QDs in comparison to sugar-free or galactose-capped-QDs.

Sialic acid-binding immunoglobulin-type lectins (SIGLECs), together with galactose-binding galectins, are cancer markers of increasing interest (Cagnoni et al., 2016). Galectin-1, 7, and 9 have been found to be overexpressed in cancer cells and therefore represent highly relevant targets in cancer diagnostics (Sun et al., 2019). A plasmonic system based on sialic acid functionalised AuNPs has been designed for the detection of SIGLECs (Schofield et al., 2016). The assay provided a colour change upon aggregation of the AuNPs in the presence of SIGLECs (in solution), or could be localised by TEM when expressed on Chinese hamster ovary cell-surface. Recently, AuNPs stabilised with acetylated mannose were used in an image-based assay to detect A549 lung cancer cells (Adokoh et al., 2020). A comparison between different mannose-functionalised NPs, revealed that those containing acetylated mannose had better selectivity towards A549 lung cancer cells than to healthy peripheral blood mononuclear cells.

García Calavia et al. reported an anti-cancer photodynamic therapy system based on bi-functionalised AuNPs (García Calavia et al., 2018). The galatose-terminal lactose disaccharide was used to selectively target galectin-1, which is overexpressed in certain breast cancer cells such as MDA-MB-231 or SK-BR-3, and a derivative of phthalocyanine was used as photosensitiser. The bi-functionalised glyco-nanoparticle conjugates could be used to achieve selective breast cancer cell death upon irradiation of the photosensitiser at 633 nm, resulting harmless to MCF-10A cells.

Human carcinoma cells have been targeted with silicon-based glyco-quantum dots (Ahire et al., 2015). Work has been done to prove that in some applications, carbohydrate-functionalised SiQDs (using galactose, mannose, glucose or lactose) are non-toxic whereas the unfunctionalised SiQDs are. Lai et al. reported an imaging system for B16F10 melanoma cells, based on low affinity carbohydrate-carbohydrate interactions between ganglioside GM3, present on the surface of cancer cells, and Gg3-functionalised 4 nm SiQDs (Lai et al., 2016). Confocal fluorescence microscopy was used to confirm that the small Gg3-SiQDs could be used to image cancer cells. A similar approach showed the uptake of glucose-functionalised 4 nm SiQDs by HeLa cells (Hsu et al., 2017), while a study of the particle size-dependent uptake of mannose-functionalised SiQDs by MDA-MB-231 breast cancer cells has been reported recently (Li et al., 2020), showing that the larger 400 nm Man-SiQDs have the highest uptake rate by cells.

Magnetic glyconanoparticles have been employed in combination with magnetic resonance imaging (MRI) to detect cancer cells, avoiding the need of labelling the cells beforehand (Ei-Boubbou et al., 2010). A collection of ferrite glyconanoparticles functionalised with either mannose, fucose, galactose, sialic acid or N-acetylglucosamine were screened against a wide variety of cancer cell lines to determine the binding preferences. In other work, AuNPs bi-functionalised with thiolated glycans (glucose, galactose or mannose) and gadolinium chelates–as paramagnetic labels for MR–were used to target hepatocytes, murine glioma cells and lymphoma cell lines (Irure et al., 2013).

A reversal approach in the functionalisation of AuNPs has been used by immobilising lectin on QDs for the detection of glycan for *in vitro* and *in vivo* imaging of tumours, together with detection and theranostic applications (Ashree et al., 2020).

## Conclusion

The range of applications of glyconanoparticles and the related carbohydrate-functionalised quantum dots is ever-expanding, with applications across detection for diagnosis of infectious diseases and cancer. For *in vivo* theranostic applications (i.e., combined diagnosis and therapy), efforts to produce more robust and less toxic nanomaterials is key to future *in vivo* applications. The field continues to progress at pace and impactful developments in the coming decade are anticipated.
